# A novel corpus of molecular to higher-order events that facilitates the understanding of the pathogenic mechanisms of idiopathic pulmonary fibrosis

**DOI:** 10.1038/s41598-023-32915-8

**Published:** 2023-04-12

**Authors:** Nozomi Nagano, Narumi Tokunaga, Masami Ikeda, Hiroko Inoura, Duong A. Khoa, Makoto Miwa, Mohammad G. Sohrab, Goran Topić, Mari Nogami-Itoh, Hiroya Takamura

**Affiliations:** 1 Artificial Intelligence Research Center, National Institute of Advanced Industrial Science and Technology (AIST), 2-4-7 Aomi, Koto-Ku, Tokyo, 135-0064 Japan; 2 Toyota Technological Institute, 2-12-1 Hisakata, Tempaku-Ku, Nagoya, 468-8511 Japan; 3 Laboratory of Bioinformatics, Artificial Intelligence Center for Health and Biomedical Research, National Institutes of Biomedical Innovation, Health and Nutrition, 3-17, Senrioka-Shinmachi, Settsu, Osaka 566-0002 Japan

**Keywords:** Pathogenesis, Literature mining, Respiratory tract diseases, Preclinical research, Molecular medicine

## Abstract

Idiopathic pulmonary fibrosis (IPF) is a severe and progressive chronic fibrosing interstitial lung disease with causes that have remained unclear to date. Development of effective treatments will require elucidation of the detailed pathogenetic mechanisms of IPF at both the molecular and cellular levels. With a biomedical corpus that includes IPF-related entities and events, text-mining systems can efficiently extract such mechanism-related information from huge amounts of literature on the disease. A novel corpus consisting of 150 abstracts with 9297 entities intended for training a text-mining system was constructed to clarify IPF-related pathogenetic mechanisms. For this corpus, entity information was annotated, as were relation and event information. To construct IPF-related networks, we also conducted entity normalization with IDs assigned to entities. Thereby, we extracted the same entities, which are expressed differently. Moreover, IPF-related events have been defined in this corpus, in contrast to existing corpora. This corpus will be useful to extract IPF-related information from scientific texts. Because many entities and events are related to lung diseases, this freely available corpus can also be used to extract information related to other lung diseases such as lung cancer and interstitial pneumonia caused by COVID-19.

## Introduction

Idiopathic pulmonary fibrosis (IPF), a severe chronic fibrosing interstitial lung disease of unclear etiology, characteristically leads to progressive and irreversible decline of lung function^1^. Idiopathic pulmonary fibrosis (IPF) acute exacerbation is a serious condition with acute respiratory failure, and representative studies have shown a 30-day survival rate of 44.6% and a 90-day survival rate of 24.6% after hospitalization for developing IPF acute exacerbation^2^. In addition, there are reports of significant fibrosis progression even after recovery, making prevention of acute exacerbations an important aspect of IPF management^3^. Although medications such as pirfenidone and nintedanib have been used to slow the progression of IPF, no medical treatment can cure IPF completely^1–5^. Pirfenidone is an antifibrotic and anti-inflammatory drug^4,6^. Nintedanib, an intracellular kinase inhibitor, targets multiple tyrosine kinases such as vascular endothelial growth factor (VEGF) receptor, fibroblast growth factor (FGF) receptor, and platelet-derived growth factor (PDGF) receptor^5^. Developing more efficient medications that can fundamentally treat the disease will necessitate elucidation of the detailed pathogenetic mechanisms of IPF at both molecular and cellular levels.

More than a hundred thousand reports of the literature on IPF have been registered in the PubMed database^7^: the most widely used online bibliographic database serving the biological sciences^8^. However, the availability of trained annotators with IPF-related knowledge is limited. Extracting adequate IPF-related information, and that of related phenomena (or ‘events’) and clinical processes, and effects of clinical treatments, from such huge amounts of information can be expected to be time-consuming. Consequently, efficient text-mining methods must be used to extract adequate information from the copious literature.

Text-mining systems have been developed for biomedical research, with information extraction algorithms and corpora corresponding particularly to systems biology, for which pathways and networks are often constructed^9^. Particularly, systems such as NERsuite^10^ and EventMine^11,12^, which employ traditional feature-based machine learning methods, have been used to extract biomedical entities and events (or phenomena) from such corpora. Recently, a neural event extraction model that employs deep learning has been proposed: DeepEventMine^13^. It shows higher performance in extracting events from such corpora. Biomedical corpora that include biomedical events have been constructed: GENIA^14,15^, Gene Regulation Event Corpus (GREC)^16^, and Cancer Genetics corpus^17–19^. In these corpora, genes and gene products (GGPs) as named entities have been annotated, along with events involving GGPs, such as gene expression and binding. Some entities and events related to IPF are annotated in the existing corpora. Nevertheless, none of these corpora are specifically associated with IPF. Information in the existing corpora is insufficient to construct IPF-related networks. Entity-linking, for which IDs must be assigned to entities, is necessary to normalize the same entities expressed differently. However, those existing corpora do not always have entity normalization. Furthermore, disease-related events have not been defined for the existing corpora, leading to difficulty in extracting disease-related events.

This work particularly examines the annotation of IPF-related entities, events, and relations to facilitate the automatic extraction of IPF-related information from scientific texts. After defining a new annotation schema for IPF-related abstracts, including the definitions of entities, events, and relations, we apply the schema and use the brat rapid annotation tool to annotate a corpus of 150 abstracts selected by experts on IPF^20,21^. Using the information in the existing corpora during the corpus development would be helpful, but the general methodologies to reuse existing corpora for the new annotation target have not been established yet. To avoid any difficulty in the annotation process, we annotate IPF-related entities, relations, and entities without relying on the existing corpora except for the automatic annotation toolkit, details of which will be described herein.

## Methods and materials

For this work, the types of entities, events, and relations, and the UMLS semantic types, which will be described below, are double-quoted. Those annotated words and phrases in text data are single-quoted, whereas event arguments, also described below, are single-quoted in italic.

### Definition of IPF-related entities

We defined essential entities involved in IPF-related phenomena and clinical processes (Table 1). Most biological entities were defined based on the GENIA meta-knowledge corpus^22,23^ and the PHAEDRA corpus^24,25^.

**Table 1:** Entity types and their occurrences.

Entity type	No. of occurrences	Frequency (per abstract)
Disorder	2090	13.93
Entity_Property	173	1.15
Measurement	136	0.91
Subject	1048	6.99
Anatomical_entity	890	5.93
Cell	813	5.42
Cell_component	25	0.17
Inorganic_compound	24	0.16
Organic_compound_other	117	0.78
Pharmacological_substance	246	1.64
GGPs	2925	19.50
Genetic_info	37	0.25
Negation_cue	74	0.49
Speculation_cue	432	2.88
Method_cue	267	1.78
Total	9297	61.98

First, the “Disorder” entity was defined to extract information related to disease, injury, and symptoms. These entities were categorized together because it is difficult and time-consuming for annotators to distinguish diseases and injuries from symptoms. “Measurement” entity was also defined for the named entity of quantification for lung diseases. For instance, ‘Forced vital capacity (FVC)’, which is measured by spirometry, can be included in this category. “Subject” was defined for patients, subjects for clinical trials, and animals used for experimentation, indicating the whole-body level.

As for the sub-whole-body level, “Anatomical_entity”, “Cell”, and “Cell_component” were defined (Table 1). Organs and tissues are categorized in “Anatomical_entity”. Entities such as ‘serum’ and ‘Bronchoalveolar Lavage Fluid’, the UMLS semantic types of which fall into “body substance”^26–28^, were also included in “Anatomical_entity” for this corpus. Cell types and cell lines are included in “Cell”. Herein, “Cell_component” is defined for cellular components such as cytoplasm, transmembranes, and organelles.

Molecular entities consist of “Pharmacological_substance”, “GGPs”, “Organic_compound_other”, and “Inorganic_compound” (Table 1). “Pharmacological_substance” is defined for medicines. “GGPs” is defined for genes or gene products. These entities were categorized together as “GGPs” because it is difficult and time-consuming for annotators to discern genes and gene products such as gene transcripts, mRNA, and proteins, in text data. Earlier, such a gene-tag annotation as “GGPs” had been proposed for other biological corpora^29,30^. “Organic_compound_other” is defined for organic compounds, excluding medicines, genes, and gene products, whereas “Inorganic_compound” denotes inorganic substances such as metal ions.

“Entity_Property” and “Genetic_info” are defined for entities that cannot be included among the entities described above (Table 1). In “Entity_Property”, other technical terms, which include the degree of disease progression/stage, cell cycle stages, and attributes, such as immunophenotyping, for cells or genes, can be categorized. Mutation information for genes is categorized as “Genetic_info”.

In addition to the entities described above, we defined cue entities “Negation_cue” and “Speculation_cue” to indicate negation or confirmation and speculation degree for events, as described below. Negation words such as ‘no’, ‘not’, and ‘none’ can be a “Negation_cue”, whereas verbs such as ‘suggest’, ‘show’, and ‘indicate’, and auxiliary verbs such as ‘may’ and ‘might’ can be included as a “Speculation_cue”. The objective of “Negation_cue” is the same as that of the Negative Polarity, which can indicate negated events, in the GENIA meta-knowledge corpus^22,23^. In addition to these two cues, “Method_cue” was defined to indicate the type of experimental study and clinical examination. “Method_cue” might also suggest confirmation and degree of speculation about an event. Named entities such as ‘CT scans’ and ‘RT-PCR’ can be categorized in this cue. These cues are usually combined with event trigger words, as described below.

### Definition of events for IPF

**Figure 1:**
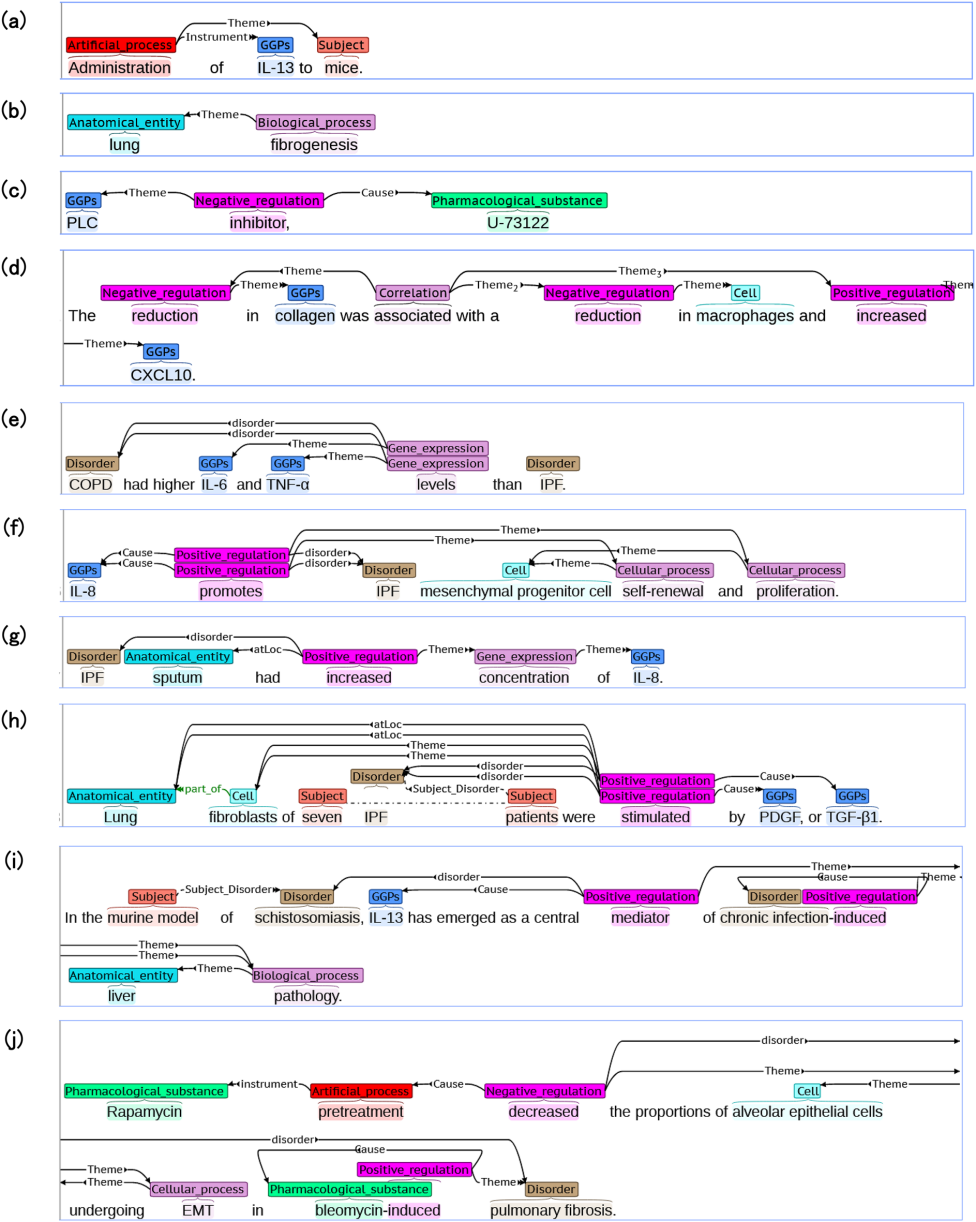
Annotation examples shown in format of brat rapid annotation tool. “Artificial_process” event (**a**), “Biological_process” event (**b**), “Negative_regulation” event (**c**), “Correlation” event with two “Negative_regulation” events and “Positive_regulation” event (**d**), ‘*disorder*’-related “Gene_expression” events (**e**), ‘*disorder*’-related “Positive_regulation” events with “Cellular_process” events (**f**), ‘*disorder*’-related “Positive_regulation” event with “Gene_expression” event (**g**), ‘*disorder*’-related “Positive_regulation” events (**h**), ‘*disorder*’-related “Positive_regulation” events with “Biological_process” event (**i**) and ‘*disorder*’-related “Negative_regulation” event with “Artificial_process” event, “Cellular_process” event and “Positive_regulation” event (**j**).

We defined artificial and biological events as presented in Table 2. Although only one artificial event was defined, biological events of several types were defined (Table 2 and Fig. 1). Most biological events were defined similarly to those in the GENIA meta-knowledge corpus^22,23^. Actually, biological events can be categorized into several events such as “Regulation”, “Correlation”, “Cellular_process”, and “Molecular_function”. Main components of these events are defined as the ‘*triggers*’ (or ‘*trigger words*’). ‘*Triggers*’ are expressed in various ways: verbal ones (e.g. ‘inhibit’), nominalizations of verbs (e.g. ‘inhibition’), and functional roles (noun) (e.g. ‘inhibitor’), in the case of ‘inhibition’ for “Negative_regulation” events. Each ‘*trigger*’ can be combined with major arguments, such as ‘*Theme*’, ‘*Cause*’, and ‘*Participant*’ along with auxiliary arguments such as ‘*atLoc*’ and ‘*disorder*’ (Table 2). In contrast to the other arguments, ‘*disorder*’ is a novel argument defined for our corpus. With the ‘*disorder*’ argument, ‘*disorder*’-related events (Fig. 1e–j) can be annotated separately from events that are not related to ‘*disorder*’ (Fig. 1a–d).

**Table 2:** Event types and their occurrences along with their argument types.

Event type	No. of occurrences	Frequency (per abstract)	Argument types
Artificial_process	368	2.45	*Theme*, *Instrument*, *disorder*
Biological_process	740	4.93	*Theme*, *Cause*, *Participant*, *Product*, *atLoc*, *fromLoc*, *toLoc*, *disorder*
Localization	229	1.53	*Theme*, *atLoc*, *fromLoc*, *toLoc*, *disorder*
Regulation	192	1.28	*Theme*, *Cause*, *atLoc*, *disorder*
Positive_regulation	1265	8.43	*Theme*, *Cause*, *atLoc*, *disorder*
Negative_regulation	570	3.80	*Theme*, *Cause*, *atLoc*, *disorder*
Correlation	335	2.23	*Theme*, *atLoc*, *disorder*
Cellular_process	241	1.61	*Theme*, *Cause*, *Participant*, *Product*, *atLoc*, *disorder*
Molecular_function	160	1.07	*Theme*, *Cause*, *Participant*, *Product*, *atLoc*, *disorder*
Conversion	61	0.41	*Theme*, *Cause*, *Product*, *atLoc*, *disorder*
Pathway	119	0.79	*Participant*, *atLoc*, *disorder*
Gene_expression	611	4.07	*Theme*, *atLoc*, *disorder*
Binding	8	0.05	*Theme*, *Product*, *atLoc*, *disorder*
Dissociation	0	0.00	*Theme*, *Product*, *atLoc*, *disorder*
Total	4899	32.66

“Regulation” events, which suggest causality (cause and effect), are classifiable into two types: “Positive_regulation”, which describes ‘activation/up-regulation’ events, and “Negative_regulation”, which describes ‘inactivation/inhibition/down-regulation’ events. However, if it is not clear whether those *trigger words* are positive or negative, the “Regulation” event will be selected. Regarding arguments for “Regulation” events, what induces these “Regulation” events can be annotated as a ‘*Cause*’ argument, whereas the effect or target can be annotated as a ‘*Theme*’ argument, as presented in Fig. 1c,f,h,i,j.

In contrast to the “Regulation” events, the “Correlation” event was also defined because causalities are unclear in many cases. When several events and entities are correlated, these “Correlation” events will be adopted. Alternatively, when several events occur simultaneously, such events can be connected with this “Correlation” event. In contrast to the “Regulation” event, more than two events or entities as ‘*Theme*’ arguments can be associated with the “Correlation” event (Fig. 1d). In the case portrayed in Fig. 1d, one “Positive_regulation” event and two “Negative_regulation” events are associated with the “Correlation” event. With these events of two types, “Regulation” and “Correlation”, the annotated entities and events can be connected to develop a network of information. The earlier reported corpus for biological events, the GENIA corpus, also includes events of both types: “Regulation” and “Correlation”^14,15,23^. However, only the “Regulation” event is defined in the Cancer Genetics corpus^17–19^.

In addition to the “Regulation” and “Correlation” events, other biological events are categorized in “Localization”, “Cellular_process”, and “Molecular_function”. Among “Molecular_function” events, more specific molecular events are further classified into “Pathway”, “Conversion”, “Gene_expression”, “Binding”, and “Dissociation”. The “Localization” event describes localization and movement of entities such as “Cell” and molecular entities including “GGPs”. The “Pathway” describes signaling transduction or metabolic pathways, where molecular entities such as “GGPs” are involved as ‘*Participant*’. The “Conversion” event describes specific reactions that involve a change in covalent bonds. ‘Phosphorylation’ is an example of a “Conversion” event. “Gene_expression” describes either transcription or translation, for which only the “GGPs” entity can be annotated as ‘*Theme*’. Although “Binding” and “Dissociation” were also defined for molecular interaction and dissociation, it turned out that there are few cases for “Binding” and none for “Dissociation” (Table 2).

Event modifications such as ‘Negated’ events and ‘Speculated’ events were also defined. The events which can be connected with “Negation_cue” are defined as ‘Negated’ events, whereas those events which can be connected with “Speculation_cue” are defined as ‘Speculated’ events. These event modifications had already been defined in other corpora such as those for Cancer Genetics and Pathway Curation^19^. Moreover, the ‘Negated’ events are the same as those ‘negated bio-events’ defined by Nawaz et al*.*^31^. They are also similar to ‘Negative polarity’ defined by Thompson et al*.*^23^.

### Normalization of entities/event triggers

The same named entities, which are often expressed differently, should be normalized to extract information properly from text data. For this work, normalization processing was performed by assigning the same ID to the same entities, which are expressed differently. Regarding such IDs, those for the Unified Medical Language System (UMLS) database (version 2018AB)^26,27^ were adopted for automatic annotation by MetaMap Lite^32,33^, which will be described below, and for the database installed in the brat annotation system^20,21^, with which the annotated IDs for UMLS were corrected manually after automatic annotation. The NCI Metathesaurus^34^, based on the UMLS database, was also used for manual annotation because the annotators had to search manually for the most appropriate terms when exact terms were not detected in the UMLS database installed in the brat system. Furthermore, event triggers were normalized along with entities.

### Definition of relations for IPF

We also defined some relations to represent static relations between entities and events. Such relations include “part_of”, “member_of”, “Subject_Disorder”, and “Disorder_association” (Table 3).

**Table 3:** Relation types and their occurrences.

Relation type	No. of occurrences	Frequency (per abstract)
part_of	460	3.07
member_of	565	3.77
Subject_Disorder	599	3.99
Disorder_association	57	0.38
Total	1681	11.21

The “part_of” relation can indicate relations of a partial entity with a whole entity, which is constituted by the partial entity. For example, this relation can indicate the relations between “Cell” and “Anatomical_entity”, such as tissues and organs. It is extremely useful to extract such relations from text data. The “member_of” relations can indicate a relation of a member with a group to which the member belongs. For example, this relation can indicate relations between a protein and its protein families, and between a patient and a patient group.

“Subject_Disorder” was defined to relate “Subject” and “Disorder”, following the relation defined in the PHAEDRA corpus^24,25^. “Disorder_association” was defined to indicate complications of diseases. Complications by two “Disorders” can be annotated by connecting the corresponding “Disorder” entities with “Disorder_association”.

### Annotation process

#### Selection of abstracts for annotation

We constructed the corpus composed of 150 abstracts of research articles on IPF-related basic research involving molecular biology. A lung disease expert manually selected the 150 abstracts: first, we narrowed down the number of IPF-related articles to about 6500 from about 100,000 articles in major journals registered in PubMed from 2013 to 2018, and selected 500 articles included in the categories of preclinical, with drugs such as pirfenidone, nintedanib, dexamethasone, tacrolimus, fluorofenidone, sirolimus, leflunomide, azithromycin, β-lapachone, sunitinib, carnosine, and tamoxifen, and without drugs. After preliminary curation to ensure that a sufficiently diverse group of molecules was included, we narrowed the list further to prioritize those with sufficiently detailed abstracts and rich descriptions: those which included descriptions of molecules and pathways associated with various respiratory diseases such as IPF and lung cancer, such as ‘TGF-β’, ‘Surfactant protein’, ‘signaling pathway’, ‘migration’, ‘macrophage’, ‘MMP’, ‘CTGF’, and ‘mucin’.

Automatic annotation, which is described in the next section, was conducted for the abstracts of the top 300 articles that were prioritized manually as described above. From the 300 automatically annotated abstracts, 120 abstracts were selected randomly for manual annotation. Moreover, from the remainder of the abstracts for inter-annotator agreement (IAA), 30 abstracts that contained numerous GGPs were selected to increase the cases of molecular events.

#### Automatic annotation by MetaMap Lite and UMLS semantic types

The MetaMap Lite 3.6.2rc3 and UMLS 2018AB datasets were applied to perform automatic annotation for the selected abstract dataset^26,27,32,33^. MetaMap Lite is a Java implementation of the basic functions of MetaMap^35,36^, which is a named entity recognition (NER) tool able to identify Unified Medical Language System (UMLS) Meta-thesaurus concepts^28^ in biomedical texts. Actually, MetaMap Lite can provide the longest concept-matched words and phrases with the UMLS concept unique identifier (CUI), designated herein as ‘UMLS ID’, as well as an “MMLite” tag. Each UMLS CUI has at least one semantic type such as “dsyn; Disease and Syndrome” and “gngm; Gene or Genome”.

The tags for the entity types, which are defined and described above, were assigned based on the semantic types. The “MMLite” tags were replaced with those tags for the entity types. For example, the “Disorder” entity tag will be assigned to the concept-matched words and phrases for the semantic type, “dsyn; Disease and Syndrome”, whereas the “GGPs” entity tag will be assigned for “gngm; Gene or Genome”. However, when a CUI (UMLS ID) is associated with multiple semantic types, selecting one automatically can engender the assignment of an unsuitable tag for the context. In such cases, annotators must consider and correct the predicted annotated entities manually.

#### Manual annotation: guideline construction and annotators

**Figure 2:**
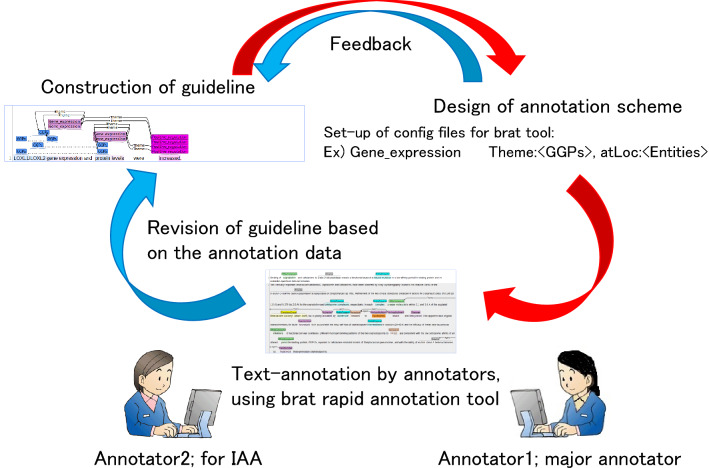
Manual annotation process for the corpus. The annotation leader constructed the annotation guideline. Based on the guideline, the annotation leader also designed the annotation scheme and the configuration for the brat tool. The annotators did text-annotation manually. The guideline was revised occasionally based on the annotation data and scheme.

The manual annotation process used for this work is presented in Fig. 2. To develop a consistent corpus, the annotation leader, a protein researcher with experience in text-annotation, constructed the annotation guideline for all annotators using the Annodoc documentation support system^37,38^. The Annodoc system is useful for constructing guidelines for text-annotation because it can readily include annotation examples in the brat format. The annotation scheme used for brat tool configuration was designed by the guideline author.

Manual annotation was conducted by an annotator (annotator 1) using the brat rapid annotation tool^20,21^. Annotator 1 has experience in the translation of biomedical documents. To ensure inter-annotator agreement (IAA), another annotator (annotator 2) performed manual annotation for 30 selected abstracts. Annotator 2 is a protein researcher specializing in signaling pathways, with experience in text-annotation for signaling pathways. The IAA dataset produced by the two annotators is available^39^.

Moreover, annotation meetings were held occasionally among the guideline author, the annotators, and the IPF expert to discuss difficult annotations. The guideline was revised based on those discussions. Also, the annotation was corrected. The guideline is available^40^.

### Evaluation

Evaluation of this corpus was based on the standard metrics of precision, recall, and F1-score. We applied the automatic entity detection and event extraction methods to the corpus and evaluated its performance. We used an event extraction system, DeepEventMine^13^, and a neural named entity recognition and linking system, BERT-based Exhaustive Neural Named Entity Recognition and Disambiguation (BENNERD)^41^.

DeepEventMine, a neural end-to-end event extraction model, extracts events from raw sentences. It performs trigger and entity recognition, relation classification, and event detection in an end-to-end manner. As another neural model, BENNERD consists of a span-based exhaustive named entity recognition model and an entity-linking model. The entity-linking model performs candidate generation that identifies a list of candidate entities in UMLS for a given mention and candidate ranking that ranks the entity candidate list to choose the best entity for the mention.

After separately evaluating event triggers and entities, entity-linking, relations and events, we used BENNERD to train individual entity recognition and linking models for each trigger and entity type. For relations, we used the trigger and entity recognition and relation extraction modules in DeepEventMine. We performed ten-fold cross-validation and measured the F1-scores with exact boundary matching for triggers, entities, and relations. For event extraction, we applied DeepEventMine, and followed the evaluation protocol adopted by BioNLP Shared Task 2009^42^ to evaluate our event prediction. In practice, we adopted the evaluation script introduced into the Cancer Genetics 2013^18^. Then we calculated the F1-scores of detected event structures using the primary matching criteria in the task.

Measurement of inter-annotator agreement (IAA) was performed using the same evaluation criteria as those used for the automatic evaluation explained above (i.e., F1 scores). We calculated the F1 scores by treating the annotations of one annotator as a gold standard and those of the other annotator as a system prediction. We switched the roles of the two annotators and averaged the F1 scores to obtain the final IAA scores. To evaluate IAA of entity-linking annotations, we only considered entities and triggers shared by the two annotators and evaluated linking annotations. Similarly, for relations, we evaluated IAA of relation types among triggers and entities shared by the two annotators to evaluate IAA based on relations alone. Regarding events, we considered entities shared by two annotators as gold entities and ignored the remaining entities and evaluated IAA.

## Results and discussion

### Tendencies in corpus contents

The corpus developed for this work was analyzed. Despite the small number of documents, only 150 abstracts, the total number of entities annotated in the corpus was 8524 (without including the three cues in Table 1), which is comparable to earlier-developed corpora such as the multi-level event extraction (MLEE) corpus, with 8291 entities^43^. Table 1 shows that “GGPs”, “Disorder”, “Subject”, “Anatomical_entity”, and “Cell” were observed frequently among all entities. The frequently observed UMLS IDs and their respective references were analyzed for the entities (Table 4).

**Table 4:** Frequently observed UMLS IDs with the UMLS reference for each entity type.

UMLS ID	UMLS: reference	No. of occurrences	Frequency per abstract	Rank
(a) Entity type: Disorder				
C1800706	Idiopathic pulmonary fibrosis	929	6.19	1
C0034069	Pulmonary fibrosis	115	0.77	2
C0012634	Disease	94	0.63	3
C0206062	Lung diseases, interstitial	93	0.62	4
C0036202	Sarcoidosis	59	0.39	5
C0016059	Fibrosis	51	0.34	6
C0024115	Lung diseases	46	0.31	7
No IDs	–	35	0.23	8
C0002390	Extrinsic allergic alveolitis	35	0.23	8
C0206061	Pneumonia, interstitial	34	0.23	9
C2350236	Idiopathic interstitial pneumonias	32	0.21	10
C0520679	Sleep apnea, obstructive	30	0.20	11
C0024117	Chronic obstructive airway disease	27	0.18	12
(b) Entity type: Measurement				
C3714541	Forced vital capacity	19	0.13	1
C1516251	Carbon monoxide diffusing capability test	17	0.11	2
C2919678	Percentage of predicted forced vital capacity	14	0.09	3
C4054207	Percent predicted diffusion capacity of the lung for carbon monoxide	13	0.09	4
C0429685	Alveolar-arterial oxygen tension difference	9	0.06	5
C0200633	Neutrophil count (procedure)	8	0.05	6
C0040509	Total lung capacity	7	0.05	7
C0042834	Vital capacity	5	0.03	8
C0202155	Oxygen measurement, partial pressure, arterial	5	0.03	8
(c) Entity type: Subject				
C0030705	Patients	555	3.70	1
C0009932	Control groups	81	0.54	2
C1257890	Population group	70	0.47	3
C0086418	Homo sapiens	65	0.43	4
C2986479	Healthy control	45	0.30	5
C0025929	Laboratory mice	39	0.26	6
C0681850	Study subject	28	0.19	7
C0001675	Adult	10	0.07	8
C1708335	Healthy volunteers	10	0.07	8
C0599755	Cohort	9	0.06	9
C2986594	Mouse model	9	0.06	9
(d) Entity type: Anatomical_entity				
C0024109	Lung	251	1.67	1
C0229671	Serum	148	0.99	2
C0006279	Bronchoalveolar lavage fluid	128	0.85	3
C0015350	Extracellular matrix	67	0.45	4
C0819757	Structure of parenchyma of lung	47	0.31	5
C0032105	Plasma	21	0.14	6
C0040300	Body tissue	20	0.13	7
C0005767	Blood	16	0.11	8
C0229664	Peripheral blood	13	0.09	9
C0038056	Sputum	10	0.07	10
C0586651	Specimen from lung obtained by biopsy	10	0.07	10
(e) Entity type: Cell				
C0016030	Fibroblasts	191	1.27	1
C0007634	Cells	95	0.63	2
C0014597	Epithelial cells	59	0.39	3
C0024432	Macrophage	42	0.28	4
C1257975	Mesenchymal stem cells	34	0.23	5
C0027950	Neutrophil	32	0.21	6
C0085236	Macrophages, alveolar	30	0.20	7
C0225360	Myofibroblasts	29	0.19	8
C0039198	Regulatory T-lymphocytes	28	0.19	9
C0225700	Type-II pneumocytes	28	0.19	9
C0039194	T-lymphocyte	27	0.18	10
C0039215	CD4 positive T lymphocytes	18	0.12	11
C0225698	Alveolar epithelial cells	18	0.12	11
(f) Entity type: Pharmacological_substance				
C0005740	Bleomycin	59	0.39	1
C2985186	FG 3019	20	0.13	2
C0001047	Acetylcysteine	19	0.13	3
C0072980	Sirolimus	11	0.07	4
C0074554	Simvastatin	10	0.07	5
C0039736	Thalidomide	9	0.06	6
C1145760	Treprostinil	9	0.06	6
C0001617	Adrenal cortex hormones	6	0.04	7
C0034392	Quercetin	6	0.04	7
C0003402	Antioxidants	5	0.03	8
C2699287	Senicapoc	5	0.03	8
C2746052	mTOR inhibitor	5	0.03	8
C2981360	Lebrikizumab	5	0.03	8
C2983747	INK128	5	0.03	8
(g) Entity type: GGPs				
C0079633	Interleukin-8	155	1.03	1
C0057628	Mucin-1 protein	99	0.66	2
No IDs	–	79	0.53	3
C0214743	Interleukin-13	70	0.47	4
C0110610	Connective tissue growth factor	69	0.46	5
C0166059	Matrix metalloproteinase 7	66	0.44	6
C0079189	cytokine	62	0.41	7
C0293060	FKBP12-rapamycin associated protein	54	0.36	8
C0017337	Genes	53	0.35	9
C1704256	Transforming growth factor beta 1	53	0.35	9
C0040690	Transforming growth factor beta	51	0.34	10
C0084692	Pulmonary surfactant-associated protein D	45	0.30	11
C0009325	Collagen	40	0.27	12

For “Disorder”, it is natural that the UMLS ID indicating ‘Idiopathic Pulmonary Fibrosis’ was the most frequently observed along with those for other lung diseases (Table 4a). In addition to these IDs for lung diseases, the UMLS ID for ‘Sarcoidosis’ was observed frequently (Table 4a). In the lungs of ‘Sarcoidosis’, the disease follows the pathology of interstitial pneumonia, and if the inflammation persists, pulmonary fibrosis may occur, limiting activity and interfering with daily life due to cough and shortness of breath. In this point of view, ‘Sarcoidosis’ appears with certain frequency. There are regional and racial differences in the incidence and severity of the disease, for example, in Europe it is more common in Northern Europe than Southern Europe^44^, and in the USA, black races are several times more susceptible and severely affected than Caucasians^45^. In Japan, by gender, twice as many women as men are detected and by age, the disease is bimodal in both men and women, in their 20 s and after their 50 s^46^. In this study, because of the focus on the respiratory tract, terms related to pulmonary fibrosis in sarcoidosis were extracted, but not terms related to the epidemiological differences described above.

Thirty-five of “Disorder” entities, which correspond to 'combined pulmonary fibrosis and emphysema', 'familial pulmonary fibrosis', 'unilateral ureteral obstruction renal fibrosis', 'non-infectious disease' and 'canine idiopathic pulmonary fibrosis', could not be assigned UMLS IDs to (Table 4a). In this corpus, 'canine idiopathic pulmonary fibrosis' was distinguished from human ‘IPF’ without being assigned the same ID.

Regarding “Measurement”, the UMLS IDs for measurements of pulmonary function and neutrophil were observed frequently (Table 4b). The UMLS IDs for ‘Patients’ and ‘Control group’ were observed most frequently for “Subject” (Table 4c).

For “Anatomical_entity”, the UMLS IDs for ‘Lung’ and ‘Serum’ were the most frequently observed, indicating that these two IDs appear once in each abstract (Table 4d). ‘Bronchoalveolar Lavage Fluid’, for which the UMLS semantic type falls into “body substance”, was also observed frequently for “Anatomical_entity” (Table 4d). Regarding “Cell”, the UMLS IDs for fibroblasts, epithelial cells, leukocytes such as neutrophils, lymphocytes, and macrophages were observed frequently (Table 4e). Although the total number of “Pharmacological_substance” is rather low (Table 1), ‘Bleomycin’, which is used to induce and model pulmonary fibrosis, medicine for IPF, ‘FG 3019’, an expectorant, ‘Acetylcysteine’, and mTOR inhibitor, such as ‘Sirolimus’, were often observed (Table 4f). Regarding “GGPs”, the UMLS IDs for cytokines and growth factors were observed frequently (Table 4g). Among the cytokines, ‘Interleukin-8’, which induces chemotaxis in target cells, was the most frequently observed (Table 4g). There were 79 “GGPs” entities with no UMLS IDs, because these entities indicate fragments, siRNA, or antibodies for some specific proteins, or ‘factors’ and ‘mediators’ that are not any specific “GGPs”, which do not have any UMLS IDs (Table 4g).

The events annotated in the corpus were 4899 (Table 2), which is a comparable number to those of some earlier developed corpora such as the MLEE corpus (6677 events)^43^, the epigenetic and post-translational modification (EPI) corpus (3714 events), and the infectious disease (ID) corpus (4150 events), which were developed by BioNLP Shared Task 2011^47^.

As shown in Table 2, “Positive_regulation” and “Biological_process” were observed most frequently among all the defined events, although the occurrences of “Binding” and “Dissociation” were very few. The frequently observed UMLS IDs and their respective references were also analyzed for event trigger words (Table 5).

**Table 5:** Frequently observed UMLS IDs with the UMLS reference for each event type.

UMLS ID	UMLS: reference	No. of occurrences	Frequency per abstract	Rank
(a) Event type: Artificial_process				
C0087111	Therapeutic procedure	63	0.42	1
C1621583	Administer	53	0.35	2
C0011900	Diagnosis	41	0.27	3
C1449619	Culture techniques	25	0.17	4
No IDs	–	12	0.08	5
C0019063	Hemoperfusion	12	0.08	5
C1522449	Therapeutic radiology procedure	11	0.07	6
C0040669	Transfection	10	0.07	7
C1516698	Collection (action)	9	0.06	8
C1535502	Bronchoalveolar lavage	9	0.06	8
C0021044	Immunohistochemistry	8	0.05	9
C0752151	Thoracic surgery, video-assisted	8	0.05	9
C0332157	Exposure to	7	0.05	10
(b) Event type: Biological_process				
C0699748	Pathogenesis	95	0.63	1
C0596570	Fibrogenesis	74	0.49	2
C0743630	Exacerbation acute	72	0.48	3
C0016059	Fibrosis	50	0.33	4
C0010957	Tissue damage	45	0.30	5
C1820201	Tissue remodeling	36	0.24	6
C0021368	Inflammation	35	0.23	7
C0242656	Disease progression	35	0.23	7
C0035245	Respiratory physiology	24	0.16	8
C1155266	inflammatory response	21	0.14	9
C0001811	Aging	20	0.13	10
C0011065	Cessation of life	20	0.13	10
C0043240	Wound healing	20	0.13	10
C0302600	Angiogenic process	20	0.13	10
(c) Event type: Localization				
C0036536	Process of secretion	53	0.35	1
C4055506	Accumulation	53	0.35	1
C1622501	Migration, cell	37	0.25	2
C1744691	Establishment and maintenance of localization	22	0.15	3
C0205234	Focal	10	0.07	4
C1692321	Cellular infiltrate	9	0.06	5
C0005528	Biological transport	6	0.04	6
C0007608	Cell motility	6	0.04	6
C0597704	Protein localization location	5	0.03	7
C0008018	Chemotaxis	4	0.03	8
C0312861	Neutrophil chemotaxis	4	0.03	8
C0007577	Cell adhesion	3	0.02	9
C3714514	Infection	3	0.02	9
(d) Event type: Cellular_process				
C0596290	Cell proliferation	54	0.36	1
C1523298	Epithelial to mesenchymal transition	31	0.21	2
C0162638	Apoptosis	18	0.12	3
C0007589	Cell differentiation process	17	0.11	4
C0004391	Autophagy	15	0.10	5
C0007620	Cell survival	11	0.07	6
C0007582	Cell communication	10	0.07	7
C0007587	Cell death	9	0.06	8
C0007595	Cell growth	9	0.06	8
C2610187	Regulation of redox homeostasis	5	0.03	9
C0007581	Cell aging	4	0.03	10
C0746885	Neutrophilic	4	0.03	10
C1516334	Cell cycle progression	4	0.03	10
(e) Event type: Molecular_function				
C0026882	Mutation	37	0.25	1
C1148560	Molecular_function	28	0.19	2
C0243102	Enzyme activity	14	0.09	3
C0032529	Genetic polymorphism	12	0.08	4
C0752046	Single nucleotide polymorphism	12	0.08	4
C0678659	Biochemical mechanism	8	0.05	5
C0599155	Missense mutation	6	0.04	6
C1158770	Transcriptional regulation	6	0.04	6
C1151115	Luciferin monooxygenase activity	5	0.03	7
C1956002	INDEL mutation	5	0.03	7
C1512032	Dominant-negative mutation	4	0.03	8
C0033666	Post-translational protein processing	3	0.02	9
C0262496	Molecular abnormality	3	0.02	9
C1150423	Kinase activity	3	0.02	9
C0162493	Transcriptional activation	2	0.01	10
C1149371	Transcription coactivator activity	2	0.01	10
C1149472	Growth factor activity	2	0.01	10
(f) Event type: Conversion				
C0031715	Phosphorylation	41	0.27	1
C0332220	Cross-linking	11	0.07	2
C0596311	Chemical cleavage	8	0.05	3
C0597304	Proteolysis	1	0.01	4
(g) Event type: Pathway				
C0037080	Signal pathways	39	0.26	1
C1706062	Metabolic networks	21	0.14	2
C1515673	mTOR signaling pathway BioCarta	8	0.05	3
C3158583	Hippo signaling	7	0.05	4
C2984399	FGF signaling pathway	6	0.04	5
C1158592	Adenosine metabolic process	5	0.03	6
C1515163	TGF beta signaling pathway BioCarta	5	0.03	6
C1622384	Adenosine receptor signaling pathway	5	0.03	6
No IDs	–	3	0.02	7
C3158959	Interleukin-13-mediated signaling pathway	3	0.02	7
C1518102	MAPK signaling pathway	2	0.01	8
(h) Event type: Gene_expression				
C1519614	Genetic translation process	350	2.33	1
C0017262	Gene expression	177	1.18	2
C0040649	Transcription, genetic	84	0.56	3

Regarding the trigger words for “Artificial_process”, the UMLS IDs for clinical actions, such as ‘Therapeutic procedure’, ‘Administer’ and ‘Diagnosis’, were most-frequently observed (Table 5a). Regarding “Biological_process”, high-order phenomena, or high-order events, such as pathogenesis, exacerbation and progression of disease, ‘Fibrosis’, and ‘Inflammation’, were observed frequently (Table 5b). ‘Exacerbation acute’ was detected as “Biological_process” event 72 times (Table 5b), of which 49 ‘*Themes*’ were IPF, for which ‘surgical lung biopsy’ of “Artificial_process” was detected as ‘*Cause*’ only once. Although the event trigger, ‘progressive respiratory failure’, was not identified in this corpus, ‘Disease Progression’ was detected 35 times, instead of such an event (Table 5b). For the ‘Disease Progression’, several “Disorder” types, and a few “Biological_process” were detected as ‘*Theme*’, among which IPF appeared 9 times. Regarding trigger words for “Localization” event, the UMLS IDs for secretion, accumulation, and cell migration were observed frequently (Table 5c). Regarding “Cellular_process”, the UMLS ID for ‘Cell Proliferation’ and ‘epithelial to mesenchymal transition (EMT)’ were observed most frequently (Table 5d). The EMT is a cellular process that engenders fibrosis, by which epithelial cells are transformed into myofibroblasts by losing cell–cell adhesion and by gaining migratory and invasive functions^48^. As trigger words for “Molecular_function” event, the UMLS ID for mutation was observed most frequently (Table 5e). For “Conversion”, the UMLS ID for ‘Phosphorylation’ was most frequently observed (Table 5f). As trigger words for “Pathway” event, the UMLS IDs for ‘Signal Pathways’ and ‘Metabolic Networks’, which are not specific networks, were observed most frequently (Table 5g). For “Gene_expression”, there are only three UMLS IDs for translation, transcription, and gene expression, among which the ID for translation was by far the most frequently observed (Table 5h).

The event arguments were also analyzed (Tables 6 and 7). Major arguments, ‘*Theme*’ and ‘*Cause*’, which are adopted by various event types, tend to take various entities and events (Table 6a,b), whereas ‘*atLoc*’, which indicates the location at which the corresponding event occurs, takes either “Anatomical_entity” or “Cell” frequently (Table 6c). Regarding the ‘*Theme*’ argument, the molecular entity “GGPs” is observed most frequently in “Localization”, “Negative_regulation”, “Correlation”, “Molecular_function”, “Conversion”, “Gene_expression”, and “Binding” (Table 6a). Molecular events such as “Molecular_function” and “Gene_expression” were also observed frequently as ‘*Theme*’ in various events (Table 6a). Regarding ‘*Cause*’, “Pharmacological_substance”, and “Organic_compound_other”, as well as “GGPs” are also observed frequently in “Positive_regulation”, and “Negative_regulation”.

**Table 6:** Frequently observed entity and event types as arguments for each event type. Molecular entities are presented in italic and bold, whereas molecular events are shown in italic.

Event type	Entity/event type	No. of occurrences	Frequency per abstract	Rank
(a) Argument type: Theme				
Artificial_process	Subject	81	0.54	1
	Cell	64	0.43	2
	Disorder	46	0.31	3
	Anatomical_entity	23	0.15	4
	***GGPs***	13	0.09	5
Biological_process	Disorder	176	1.17	1
	Anatomical_entity	112	0.75	2
	Cell	44	0.29	3
	Subject	18	0.12	4
	Biological_process	14	0.09	5
Localization	***GGPs***	108	0.72	1
	Cell	85	0.57	2
	Anatomical_entity	16	0.11	3
Regulation	Biological_process	40	0.27	1
	*Gene_expression*	27	0.18	2
	Cell	25	0.17	3
	Positive_regulation	24	0.16	4
	Cellular_process	14	0.09	5
	***GGPs***	13	0.09	6
	*Pathway*	13	0.09	6
	Disorder	10	0.07	7
Positive_regulation	*Gene_expression*	264	1.76	1
	***GGPs***	224	1.49	2
	Cell	204	1.36	3
	Biological_process	167	1.11	4
	Cellular_process	96	0.64	5
	Disorder	88	0.59	6
	Localization	75	0.50	7
	Positive_regulation	63	0.42	8
	Negative_regulation	38	0.25	9
	*Conversion*	29	0.19	10
	*Pathway*	26	0.17	11
	*Molecular_function*	17	0.11	12
Negative_regulation	***GGPs***	126	0.84	1
	*Gene_expression*	108	0.72	2
	Biological_process	84	0.56	3
	Cell	67	0.45	4
	Cellular_process	49	0.33	5
	Localization	37	0.25	6
	Positive_regulation	24	0.16	7
	Measurement	22	0.15	8
	Disorder	21	0.14	9
	*Molecular_function*	18	0.12	10
	*Conversion*	10	0.07	11
Correlation	***GGPs***	171	1.14	1
	Disorder	147	0.98	2
	Biological_process	132	0.88	3
	Cell	68	0.45	4
	Measurement	58	0.39	5
	Positive_regulation	53	0.35	6
	Cellular_process	33	0.22	7
	Negative_regulation	32	0.21	8
	Localization	27	0.18	9
	*Molecular_function*	25	0.17	10
	*Pathway*	24	0.16	11
	*Gene_expression*	18	0.12	12
	Regulation	13	0.09	13
Cellular_process	Cell	100	0.67	1
Molecular_function	***GGPs***	109	0.73	1
Conversion	***GGPs***	50	0.33	1
Gene_expression	***GGPs***	593	3.95	1
Binding	***GGPs***	14	0.09	1
(b) Argument type: Cause				
Biological_process	***GGPs***	35	0.23	1
Regulation	***GGPs***	72	0.48	1
	***Pharmacological_substance***	34	0.23	2
	Cell	13	0.09	3
	Negative_regulation	12	0.08	4
Positive_regulation	***GGPs***	376	2.51	1
	Positive_regulation	61	0.41	2
	***Pharmacological_substance***	51	0.34	3
	Disorder	44	0.29	4
	Artificial_process	39	0.26	5
	***Organic_compound_other***	36	0.24	6
	Biological_process	34	0.23	7
	Cell	32	0.21	8
	Negative_regulation	24	0.16	9
	*Pathway*	18	0.12	10
	*Gene_expression*	16	0.11	11
	Cellular_process	13	0.09	12
	*Molecular_function*	10	0.07	13
Negative_regulation	***Pharmacological_substance***	147	0.98	1
	***GGPs***	75	0.50	2
	Negative_regulation	61	0.41	3
	Artificial_process	16	0.11	4
	Cell	11	0.07	5
	***Organic_compound_other***	11	0.07	5
Molecular_function	***GGPs***	11	0.07	1
Conversion	***GGPs***	11	0.07	1
(c) Argument type: atLoc				
Biological_process	Anatomical_entity	25	0.17	1
Localization	Cell	34	0.23	1
	Anatomical_entity	28	0.19	2
Positive_regulation	Anatomical_entity	102	0.68	1
	Cell	40	0.27	2
Negative_regulation	Cell	15	0.10	1
	Anatomical_entity	8	0.05	2
Cellular_process	Anatomical_entity	10	0.07	1
Molecular_function	Cell	6	0.04	1
	Anatomical_entity	4	0.03	2
Gene_expression	Cell	182	1.21	1
	Anatomical_entity	56	0.37	2

**Table 7:** Frequently observed UMLS IDs as arguments for each event type. Molecular entities for UMLS reference are presented in italic and in bold, whereas molecular events are shown in italic.

Event type	UMLS ID	UMLS: reference	No. of occurrences	Frequency per abstract	Rank
(a) Argument type: Theme					
Artificial_process	C0030705	Patients	36	0.24	1
	C1800706	Idiopathic pulmonary fibrosis	29	0.19	2
	C0016030	Fibroblasts	23	0.15	3
	C0007634	Cells	17	0.11	4
	C0025929	Laboratory mice	17	0.11	4
Biological_process	C1800706	Idiopathic pulmonary fibrosis	90	0.60	1
	C0024109	Lung	56	0.37	2
	C0014597	Epithelial cells	19	0.13	3
	C0015350	Extracellular matrix	18	0.12	4
	C0034069	Pulmonary fibrosis	17	0.11	5
	C0030705	Patients	14	0.09	6
	C0206062	Lung diseases, interstitial	12	0.08	7
Localization	C0079633	***Interleukin-8***	24	0.16	1
	C0016030	Fibroblasts	18	0.12	2
	C0027950	Neutrophil	16	0.11	3
	C0015350	Extracellular matrix	13	0.09	4
	C0009325	***Collagen***	10	0.07	5
Regulation	C1879547	Activation action	24	0.16	1
	C1519614	*Genetic translation process*	16	0.11	2
	C0699748	Pathogenesis	12	0.08	3
	C0017262	*Gene expression*	10	0.07	4
Positive_regulation	C1519614	*Genetic translation process*	159	1.06	1
	C0017262	*Gene expression*	70	0.47	2
	C1879547	Activation action	63	0.42	3
	C3463820	Inhibition	38	0.25	4
	C0040649	*Transcription, genetic*	35	0.23	5
	C0016030	Fibroblasts	32	0.21	6
	C0034069	Pulmonary fibrosis	29	0.19	7
	C1622501	Migration, cell	26	0.17	8
	C0031715	*Phosphorylation*	24	0.16	9
	C0596290	Cell proliferation	23	0.15	10
Negative_regulation	C1519614	*Genetic translation process*	48	0.32	1
	C0017262	*Gene expression*	42	0.28	2
	C0596570	fibrogenesis	24	0.16	3
	C1879547	Activation action	24	0.16	3
	C0035245	Respiratory physiology	19	0.13	4
	C0040649	*Transcription, genetic*	18	0.12	5
	C0293060	***FKBP12-rapamycin associated protein***	14	0.09	6
	C1622501	Migration, cell	12	0.08	7
	C0034069	Pulmonary fibrosis	11	0.07	8
	C0036536	Process of secretion	11	0.07	8
Correlation	C1800706	Idiopathic pulmonary fibrosis	59	0.39	1
	C1879547	Activation action	53	0.35	2
	C3463820	Inhibition	32	0.21	3
	C0699748	Pathogenesis	28	0.19	4
	C0016059	Fibrosis	22	0.15	5
	C0079633	***Interleukin-8***	20	0.13	6
	C4055506	Accumulation	16	0.11	7
	C0017337	***Genes***	14	0.09	8
	C1327622	Regulation of biological process	13	0.09	9
	C0012634	Disease	12	0.08	10
	C0034069	Pulmonary Fibrosis	12	0.08	10
	C0057628	***Mucin-1 protein***	12	0.08	10
Gene_expression	C0079633	***Interleukin-8***	36	0.24	1
	C0110610	***connective tissue growth factor***	36	0.24	1
	no UMLS ID	–	19	0.13	2
	C0172956	***Neutrophil Collagenase***	19	0.13	2
	C0017337	***Genes***	14	0.09	3
	C0079189	***cytokine***	10	0.07	4
	C1456820	***Tumor Necrosis Factor-alpha***	10	0.07	4
(b) Argument type: Cause					
Biological_process	C0005740	***Bleomycin***	7	0.05	1
	C0282554	***chemokine***	7	0.05	1
	C0079189	***cytokine***	6	0.04	2
Regulation	C0001047	***Acetylcysteine***	14	0.09	1
	C3463820	Inhibition	12	0.08	2
	C0214743	***Interleukin-13***	8	0.05	3
	C2985186	***FG 3019***	8	0.05	3
	C0527729	***Interleukin-13 Receptor alpha1 Subunit***	6	0.04	4
	C0079633	***Interleukin-8***	5	0.03	5
	C1145760	***Treprostinil***	5	0.03	5
Positive_regulation	C1879547	Activation action	61	0.41	1
	C0005740	***Bleomycin***	30	0.20	2
	C0214743	***Interleukin-13***	29	0.19	3
	C0670902	***Tumor Necrosis Factor Ligand Superfamily Member 14***	29	0.19	3
	C1704256	***Transforming Growth Factor Beta 1***	29	0.19	3
	C0242184	Hypoxia	26	0.17	4
	C3463820	Inhibition	24	0.16	5
	C0079633	***Interleukin-8***	23	0.15	6
	C1621583	Administer	20	0.13	7
	C0023810	***Lipopolysaccharides***	18	0.12	8
	C0040690	***Transforming Growth Factor beta***	17	0.11	9
	C0218504	***Chemokine CXCL12***	15	0.10	10
Negative_regulation	C3463820	Inhibition	61	0.41	1
	C0001047	***Acetylcysteine***	26	0.17	2
	C0039736	***Thalidomide***	19	0.13	3
	no UMLS ID	–	18	0.12	4
	C1099354	***RNA, Small Interfering***	15	0.10	5
	C2985186	***FG 3019***	14	0.09	6
	C0074554	***Simvastatin***	10	0.07	7
	C1145760	***Treprostinil***	10	0.07	7
	C2983747	***INK128***	9	0.06	8
	C0087111	Therapeutic procedure	7	0.05	9
	C0214743	***Interleukin-13***	7	0.05	9
	C2746052	***mTOR Inhibitor***	7	0.05	9
	C0127082	***Interstitial Collagenase***	5	0.03	10
	C1707080	***temsirolimus***	5	0.03	10
Conversion	C0166059	***Matrix Metalloproteinase 7***	6	0.04	1
(c) Argument type: disorder					
Artificial_process	C1800706	Idiopathic pulmonary fibrosis	17	0.11	1
Biological_process	C1800706	Idiopathic pulmonary fibrosis	19	0.13	1
Localization	C1800706	Idiopathic pulmonary fibrosis	8	0.05	1
Regulation	C1800706	Idiopathic pulmonary fibrosis	5	0.03	1
Positive_regulation	C1800706	Idiopathic pulmonary fibrosis	154	1.03	1
	C0036202	Sarcoidosis	21	0.14	2
	C0002390	Extrinsic allergic alveolitis	11	0.07	3
	C0034069	Pulmonary fibrosis	9	0.06	4
	C0024117	Chronic obstructive airway disease	5	0.03	5
Negative_regulation	C1800706	Idiopathic pulmonary fibrosis	34	0.23	1
	C0034069	Pulmonary fibrosis	5	0.03	2
	C0206062	Lung diseases, interstitial	5	0.03	2
Correlation	C1800706	Idiopathic pulmonary fibrosis	12	0.08	1
Cellular_process	C1800706	Idiopathic pulmonary fibrosis	6	0.04	1
Molecular_function	C1800706	Idiopathic pulmonary fibrosis	9	0.06	1
Gene_expression	C1800706	Idiopathic pulmonary fibrosis	66	0.44	1
	C0034069	Pulmonary fibrosis	7	0.05	2
(d) Argument type: atLoc					
Biological_process	C0024109	Lung	17	0.11	1
Localization	C0024109	Lung	11	0.07	1
Positive_regulation	C0024109	Lung	28	0.19	1
	C0006279	Bronchoalveolar lavage fluid	20	0.13	2
	C0229671	Serum	14	0.09	3
	C0016030	Fibroblasts	12	0.08	4
	C1550101	Supernatant	12	0.08	4
Gene_expression	C0016030	Fibroblasts	53	0.35	1
	C0007634	Cells	35	0.23	2
	C0085236	Macrophages, alveolar	34	0.23	3
	C0024109	Lung	22	0.15	4
	C0024432	Macrophage	11	0.07	5

The frequently observed UMLS IDs were also analyzed for the arguments (Table 7). The UMLS ID for ‘IPF’ was observed most frequently as ‘*Theme*’ in two events: “Biological_process” and “Correlation” (Table 7a). In comparison with ‘*Theme*’, the UMLS IDs for various molecules are observed frequently as ‘*Cause*’ in various events, “Biological_process”, “Regulation”, “Positive_regulation”, “Negative_regulation”, and “Conversion” (Table 7b). It is natural that the UMLS ID for ‘IPF’ was the most frequently observed as ‘*disorder*’ in various events (Table 7c). It is also natural that the UMLS ID for ‘Lung’ is observed frequently as ‘*atLoc*’ in various events (Table 7d).

### Evaluation results by ten-fold cross-validation

Using ten-fold cross-validation, named entity recognition (NER), entity-linking, event extraction, and relation extraction were conducted to evaluate this corpus. Cross-validation is aimed at evaluating the corpus consistency, and also at examining how much state-of-the-art text-mining systems can address these tasks in the corpus.

Overall F1 scores for entities and event triggers by NER were, respectively, 87.43 and 84.40 (Table 8), which indicates that this corpus can contribute to text-mining for IPF research in terms of NER. However, F1 scores for “Genetic_info”, “Inorganic_compound”, “Cell_component”, and “Binding”, for which the occurrences were very few, are lower than 50.0 (Table 8a). Particularly, the F1 score for “Binding” was zero because the number of occurrences is only eight (Tables 1 and 8a). The F1 scores of NER are correlated with the number of occurrences (Tables 1, 2, and 8) (correlation coefficients were 0.62 for entities and 0.53 for event triggers). Moreover, because a small number of entities and event triggers cannot be distributed equally in all folds in ten-fold cross-validation, some folds contain no such entities and event triggers, which engender zero precision, recall, and F1. Such deviations of the distribution are apparently negatively correlated with the F1 scores. From more specific viewpoints of event triggers, the F1 scores for event triggers of “Regulation” and “Correlation”, 61.96 and 75.26, respectively, are much lower than those of “Positive_regulation” and “Negative_regulation”, 91.61 and 92.35, respectively (Table 8b). Because it is difficult to distinguish event triggers for “Regulation” and “Correlation” from those for “Positive_regulation”, the performance of “Regulation” and “Correlation” might be lower. Regarding IAA measurement, the IAA score for NER of entities and cues shows 79.42, whereas that of event triggers shows 71.31. These IAA scores are lower than the F1 scores for NER by ten-fold cross-validation (87.43 for entities and cues; 84.40 for event triggers) (Table 8).

**Table 8:** Evaluation of entities and event triggers by named entity recognition (ten-fold cross validation).

Entity/event type	Precision	Recall	F1
(a) Named entity recognition of each entity			
Disorder	91.72	91.44	91.53
Entity_Property	65.95	69.22	63.86
Measurement	67.85	76.01	69.78
Subject	86.98	87.74	87.28
Anatomical_entity	89.17	91.43	90.19
Cell	89.12	89.40	89.14
Cell_component	50.00	42.50	45.24
Inorganic_compound	38.75	37.08	37.84
Organic_compound_other	74.65	46.88	52.43
Pharmacological_substance	84.84	91.04	87.22
GGPs	89.20	92.33	90.70
Genetic_info	20.00	12.93	15.24
Negation_cue	63.28	64.65	61.76
Speculation_cue	67.41	74.19	70.50
Method_cue	76.43	80.96	78.34
Overall	86.74	88.19	87.43
(b) Named entity recognition of each event trigger			
Artificial_process	77.01	74.76	75.51
Biological_process	78.73	82.24	80.33
Localization	91.69	86.97	88.92
Regulation	61.44	64.38	61.96
Positive_regulation	90.95	92.46	91.61
Negative_regulation	90.76	94.25	92.35
Correlation	74.29	78.11	75.26
Cellular_process	86.00	82.32	83.00
Molecular_function	77.43	67.96	70.72
Conversion	78.89	65.72	70.42
Pathway	85.36	69.37	75.53
Gene_expression	90.03	94.16	92.01
Binding	0.00	0.00	0.00
Overall	84.49	84.38	84.40

Results of entity-linking for ten-fold cross-validation are presented in Table 9. As a whole, the performance of entity-linking for entities is good: the F1 score of entity-linking for entities is 68.21 (Table 9a). Because the UMLS IDs for “Genetic_info”, “Negation_cue”, and “Speculation_cue” are not annotated, these data are not included in Table 9a. The F1 scores for “Cell_component” and “Inorganic_compound”, for which the numbers of occurrences were fewer than 30, were lower than 30. The F1 scores of entity-linking for entities correlate with the numbers of occurrences for entities (Tables 1 and 9a) (correlation coefficient, 0.52). However, the F1 score of entity-linking for event triggers is 58.21 (Table 9b), which is lower than that of the entities. The F1 scores for “Regulation”, “Conversion”, “Pathway”, and “Binding” were lower than 30. Particularly, the F1 score for “Binding” was 0.00. Regarding “Conversion”, “Pathway”, and “Binding”, it seems natural that the F1 scores are very low because their occurrences were fewer than 150 (Table 2). The F1 scores of entity-linking for event triggers correlate with the numbers of occurrences for event triggers (Tables 2 and 9b) (correlation coefficient, 0.81), and also with the F1 scores for event triggers in NER (Tables 8b and 9b) (correlation coefficient, 0.73). Regarding the IAA measurement, the IAA score for entity-linking for entities is 72.27, which is lower than that of NER for entities and cues (79.42). However, the IAA score for entity-linking for event triggers is 84.08, which is much higher than that of NER for event triggers (71.31). In contrast to the IAA scores for NER, these IAA scores are higher than the F1 scores for entity-linking by ten-fold cross-validation (68.21 for entities and cues; 58.21 for event triggers) (Table 9).

**Table 9:** Evaluation by entity-linking (ten-fold cross validation).

Entity/event type	Precision	Recall	F1
(a) Entities			
Disorder	88.33	77.16	82.21
Entity_Property	62.04	22.74	29.65
Measurement	38.09	32.40	34.82
Subject	81.57	67.32	73.62
Anatomical_entity	80.72	76.24	78.29
Cell	78.33	69.12	73.18
Cell_component	26.67	25.00	25.71
Inorganic_compound	0.00	0.00	0.00
Organic_compound_other	73.75	38.77	47.99
Pharmacological_substance	84.91	63.93	72.05
GGPs	62.27	54.08	57.81
Method_cue	62.37	51.95	56.39
Overall	74.06	63.32	68.21
(b) Event triggers			
Artificial_process	49.53	38.37	43.03
Biological_process	65.07	49.82	56.23
Localization	82.44	45.42	57.71
Regulation	54.17	14.73	*22.15*
Positive_regulation	91.50	79.45	84.85
Negative_regulation	90.87	67.94	77.16
Correlation	64.24	43.68	51.11
Cellular_process	41.92	31.26	35.13
Molecular_function	44.24	29.76	34.59
Conversion	50.00	17.44	24.95
Pathway	24.90	14.48	17.30
Gene_expression	38.99	37.12	37.90
Binding	0.00	0.00	0.00
Overall	68.48	50.73	58.21

Results of event extraction and relation extraction, which usually exhibits worse performance than NER in any corpus, are presented in Table 10. The F1 score of event extraction is 45.08: markedly lower than 50 (Table 10a). As a whole, F1 scores of events tend to be lower than 50.0, although those for “Biological_process”, “Cellular_process”, and “Gene_expression” are approximately 60.0, which is higher than the other events (Table 10a). In the MLEE corpus^43^, the F score for event extraction of anatomical events, which correspond to “Biological_process” and “Cellular_process” in our corpus, is the highest among all the events, suggesting that these events are readily extracted. The F1 scores of the event extraction are not so correlated with the number of occurrences (Tables 2 and 10a) (correlation coefficient, 0.33), but correlated with the F1 scores of event triggers in NER (Tables 8b and 10a) (correlation coefficient, 0.75). However, although the F1 scores of NER event triggers for “Positive_regulation” and “Negative_regulation” are very high (91.61 and 92.35, respectively) (Table 8b), those F1 scores of event extraction are rather low (35.97 and 41.11, respectively) (Table 10a). Generally, the performance of event extraction for such regulation events is lower than those for other events, considering other corpora such as the Cancer Genetics (CG) corpus and the Pathway corpus^17–19^, and the GENIA corpus^49^. In comparison with the F1 scores of event extraction for the MLEE corpus and the CG corpus using DeepEventMine^13,50^, the F1 scores of this corpus tend to be lower than these previous corpora, probably due to the larger number of arguments and increased degree of expressions for trigger words. For instance, in the case of “Gene_expression”, F1 score of this corpus showed 59.34, whereas those scores of the MLEE and the CG corpora were 80.80 and 82.64, respectively^50^. In the case of “Pathway”, F1 score of this corpus showed 54.01, whereas those of the MLEE and the CG corpora were 69.33 and 73.54, respectively^50^. By introducing a new argument, ‘*disorder*’, the event structures for this corpus became even more complicated. Moreover, the regulation events often include other events as arguments (‘*Theme*’ and ‘*Cause*’) recursively, which might make their extraction challenging^19^. Thus, it will be necessary to develop a new event extraction system that can extract such complicated events more efficiently and correctly in the future. The IAA score for event extraction is 53.42, which is higher than that for event extraction by ten-fold cross-validation (45.08) (Table 10a). Moreover, the IAA score for event extraction is much lower than any other IAA score. This lower score suggests that event annotation is most difficult to carry out consistently. It also requires more trained annotation skills than any other annotation, such as entities, normalization (ID assignment) and relations, because event structures are the most complicated with event triggers and their relations with several arguments. Because this corpus dataset was annotated by only one annotator (annotator 1), it is largely free of inconsistencies that are unavoidable in a dataset constructed by multiple annotators, especially in terms of event annotation.

**Table 10:** Evaluation by event extraction and relation extraction (ten-fold cross validation).

Event type	Precision	Recall	F1
(a) Event extraction			
Artificial_process	40.61	32.34	35.56
Biological_process	61.47	58.62	59.97
Localization	59.53	43.00	49.56
Regulation	40.38	23.67	27.15
Positive_regulation	43.77	30.63	35.97
Negative_regulation	47.16	36.99	41.11
Correlation	38.40	18.84	24.54
Cellular_process	73.61	58.54	64.56
Molecular_function	38.49	28.05	31.65
Conversion	37.00	17.81	23.65
Pathway	63.07	48.49	54.01
Gene_expression	57.28	61.85	59.34
Binding	0.00	0.00	0.00
Overall	51.55	40.09	45.08

Modification type	Precision	Recall	F1
(b) Event extraction for event modifications			
Negated	35.86	20.08	25.64
Speculated	51.71	26.98	34.92
Overall	51.59	26.09	34.24

Relation type	Precision	Recall	F1
(c) Relation extraction			
Part_of	40.32	38.28	38.54
Member_of	42.81	34.34	36.73
Subject_Disorder	65.24	71.16	67.33
Disorder_association	56.98	42.75	45.19
Overall	51.18	48.80	49.64

The F1 score of event extraction for event modification is 34.24, which is even lower than that of the event extraction above (Table 10b). The F1 score of ‘Negated’ events is 25.64, whereas that of ‘Speculated’ events is 34.92. Regarding ‘Negated’ events, the instances of ‘Negated’ in the gold data are only 93, which can be a reason why its performance is very low. Furthermore, in the other corpora, such as those for Cancer Genetics and Pathway Curation, the event extraction for event modification was apparently challenging, with F1 scores of approximately 30^19^.

The F1 score of relation extraction is 49.64, also lower than 50, but slightly better than that of event extraction, probably because the relation models are much simpler than the event models. The F1 scores for “Subject_Disorder” and “Disorder_association” are higher than 40, whereas those for “part_of” and “member_of” are lower than 40. The F1 scores of the relation extraction are not so correlated with the number of occurrences (Tables 3 and 10c) (correlation coefficient, 0.23). The related entities for “Subject_Disorder” and “Disorder_association” are very specific, which might make their extraction easier. In contrast, the relations represented by “part_of” and “member_of” are rather complicated, involving various entity types, which might make the extraction more difficult. The IAA score for relation extraction is 76.35, which is much higher than that by ten-fold cross-validation (49.64) (Table 10c).

### Novelty and significance of the corpus

To extract and construct a network that is related to the disorder, IPF, entity-linking and annotation data of IPF-related events are necessary. Because many entities are expressed differently, extracted entities should be assigned with IDs so that the same entities can be matched in the networks. Entity-linking in this corpus enables this ID assignment for entities.

Regarding the IPF-related events, those existing corpora cannot provide ‘*disorder*’-related event data. In this corpus, ‘*disorder*’-related events have been annotated as indicated in Fig. 1 (Fig. 1e–j). Combined with this corpus, state-of-the-art text-mining system might be able to extract ‘*disorder*’-related events that are distinguishable from the other ordinary events (Fig. 1a–d) in the near future.

Moreover, this corpus encompasses multiple levels of organisms from molecular level to the whole body level. As an existing corpus for multiple levels of organisms, the MLEE corpus, which has emphasized angiogenesis, the development of new blood vessels, has been reported^43^. The types of entities and events in our corpus were compared with those of the MLEE corpus (Table 11). Most of the MLEE entities correspond to the entities in our corpus, except for “PROTEIN DOMAIN OR REGION” and “DNA DOMAIN OR REGION”, which are not defined in our corpus (Table 11a). In our corpus, a molecular entity, “DRUG OR COMPOUND”, of the MLEE corpus was subdivided into the three entities, “Inorganic_compound”, “Organic_compound_other”, and “Pharmacological_substance”. In contrast, various anatomical entities of the MLEE corpus, such as “ANATOMICAL SYSTEM”, “ORGAN”, “MULTI-TISSUE STRUCTURE”, and “TISSUE” are integrated into one entity, “Anatomical_entity”, in our corpus. Although preclinical text data were targeted in our corpus, clinical terms, especially for pulmonary disorders, are annotated in “Measurement”, “Entity_property”, and “Method_cue”, which have not been annotated in the MLEE corpus. With these clinical entities, NER and entity-linking can be performed for the clinical literature on lung diseases.

**Table 11:** Entity/event types in this corpus and those defined in the MLEE corpus.

Entity/event type in this corpus	MLEE entity/event	Category of MLEE entities/events
(a) Entity types		
Disorder	Pathological formation	Anatomy
Entity_Property		
Measurement		
Subject	Organism	Organism
Anatomical_entity	Organism subdivision; anatomical system; organ; multi-tissue structure; tissue; developing anatomical structure; organism substance; immaterial anatomical entity	Anatomy
Cell	Cell	Anatomy
Cell_component	Cellular component	Anatomy
Inorganic_compound	*Drug or compound*	Molecule
Organic_compound_other	*Drug or compound*	Molecule
Pharmacological_substance	*Drug or compound*	Molecule
GGPs	Gene or gene product	Molecule
Genetic_info		
	Protein domain or region; DNA domain or region	Molecule
Negation_cue		
Speculation_cue		
Method_cue		
(b) Event types		
Artificial_process	Planned process	Planned
Biological_process	Development; blood vessel development; growth; death; breakdown; remodeling; reproduction	Anatomical
Localization	Localization	General
Regulation	Regulation	General
Positive_regulation	Positive_regulation	General
Negative_regulation	Negative_regulation	General
Correlation		
Cellular_process	Cell proliferation; cell division	Anatomical
Molecular_function	metabolism; synthesis; catabolism	Molecular
Conversion	Phospholylation; dephospholylation; acetylation; ubiquitination; DNA methylation	Molecular
Pathway	Pathway	Molecular
Gene_expression	Gene expression; transcription; translation	Molecular
Binding	Binding	General
Dissociation	Dissociation	General

All MLEE events correspond to events in our corpus (Table 11b). At the cellular level, the MLEE corpus has emphasized “CELL PROLIFERATION” and “CELL DIVISION.” In contrast, the wider scope of the cellular events, including EMT, autophagy and cell communication, has been covered in our corpus (Table 5d). At the anatomical level, the MLEE corpus has mainly emphasized “BLOOD VESSEL DEVELOPMENT”, “DEVELOPMENT”; and angiogenesis-related events, such as “GROWTH” “DEATH”, “BREAKDOWN”, and “REMODELING”, whereas fibrosis-related events such as fibrogenesis, fibrosis, and inflammation, have been annotated more intensively in our corpus (Table 5b). In this corpus, molecules involved in inflammation and fibrosis are listed. For example, molecules involved in NFκB signaling and integrin signaling which are related to inflammatory cytokines^51^. As related to fibrosis, molecules such as TGFβ, surfactant proteins and molecules involved in the Wnt-β catenin signaling are also included^51,52^. The background of pathological process from inflammation to fibrosis can be understood by discovering the relationships and regulatory relations among these molecules. With these differences from the MLEE corpus, our corpus can emphasize the pulmonary disorder-related events and can facilitate extraction of these events.

Although the reuse of the existing corpora in the annotation and evaluation were not addressed in this study because reuse is beyond the scope of our study, the existing corpora can facilitate improvement of the performance of the disorder-related event extraction by combining our corpus with the existing corpora. We leave this as a subject for future work.

## Conclusion

We have presented a new corpus for molecular and cellular mechanisms for a chronic fibrosing interstitial lung disease, idiopathic pulmonary fibrosis (IPF)^53^. The corpus is expected to be useful to extract IPF pathogenesis mechanisms automatically from huge amounts of scientific texts. We defined entities, events, and relations, annotated a corpus of 150 abstracts, and applied existing state-of-the-art NER and event extraction systems to the corpus. By obtaining timely molecular information from previous reports, we can find the missing links in the previous findings using this corpus combined with the recent text-mining systems. Thus, we will extract molecules related to the acute exacerbation and progressive respiratory failure, or molecules related to inflammation and fibrosis, and furthermore, we will draw their relationship. Moreover, we can find the upstream regulatory molecules of the extracted molecules. We believe that these analyses will help in the search for therapeutic methods. Although this corpus has emphasized IPF, it is applicable to the extraction of information related to other lung diseases, including lung cancer and interstitial pneumonia caused by COVID-19 because some entities and events of this corpus are related also to such diseases.

## Data Availability

The following datasets are freely available at their respective websites. The corpus for IPF pathogenetic mechanisms: https://ezcatdb.github.io/prism_IPFdata/IPF_corpus/. IAA dataset. Data by annotator 1: https://ezcatdb.github.io/prism_IPFdata/iaa/iaa_1/. Data by annotator 2: https://ezcatdb.github.io/prism_IPFdata/iaa/iaa_2/. Annotation guideline for this work: https://ezcatdb.github.io/prism_IPFdata/AnnotationGuideline_IPFmechanism.pdf.

## Abbreviations

BENNERD: BERT-based exhaustive neural named entity recognition and disambiguation
BERT: Bidirectional encoder representations from transformers
BioNLP: Biomedical natural language processing workshop
COVID-19: Coronavirus disease 2019
CT: Computed tomography
CTGF: Connective tissue growth factor
CUI: Concept unique identifier
EMT: Epithelial to mesenchymal transition
EPI: Epigenetic and post-translational modification
FGF: Fibroblast growth factor
FVC: Forced vital capacity
GGPs: Gene and gene products
GREC: Gene regulation event corpus
IAA: Inter-annotator agreement
IPF: Idiopathic pulmonary fibrosis
MLEE: Multi-level event extraction
MMLite: MetaMap Lite
MMP: Matrix metalloproteinase
mTOR: Mammalian target of rapamycin
NCI: US National Cancer Institute
NER: Named entity recognition
PDGF: Platelet-derived growth factor
RT-PCR: Reverse transcription polymerase chain reaction
TGF: Transforming growth factor
UMLS: Unified medical language system
VEGF: Vascular endothelial growth factor
